# Red Noses

**Published:** 1890-12

**Authors:** 


### RED NOSES.

The following is from the *Christian Union:* A woman is getting too much complexion on her nose, just where she doesn't want it, and too much development upon her abdomen, just where she abominates it, all through tight lacing, then and not until then will she let out her corsets and wear them as she should. You see, the philosophy of it is this : Tight corsets produce just the internal derangements which result in undue redness of the nose, and besides, they retard the circulation. Any thing which tends to send the blood to the head in undue quantities affects the circulation of the face, and the nose, being the most prominent member, is the first to hang out the danger signal, to the distress of its owner. The multitudinous and infinitesimal veins and cells, distended in an unnatural manner the greater part of the time, ultimately go off on a strike, with the unreasonableness of other hard laborers, and refuse to perform their work of contraction even when all obstacles are removed and hours are shortened to their liking ; and this results in a permanently glowing member, causing great annoyance and vexation. Of course in a fleshy nose the result is most disastrous, as the circulation is more apparent, while if the feature is cartilaginous, there is little or no blood circulation, and the effect is not quite so palpable and distressing.

You see, I've been in the business a long time, first with regular corsets, now with these waists. I remember that the pretty young girls who used to insist on my pulling up their corsets until the new laces snapped like threads, came back to me in ten years with sallow skins, pale lips, and dull eyes. Liver gets all wrong, you see, and when your liver gets wrong every thing is in a jaundiced condition. Marriage is a failure, and life is a burden, and heaven is a myth. I know a lady who will admit, now that she has really reformed, that three weeks of tight lacing will thicken her skin like a piece of parchment, and make her believe that her husband is in love with some one else, her cook steals the sugar, her diamonds are paste, and her baby is going to die if it cries with wind on its stomach. I could tell you, too, of the girls oculists send here because the wearing of tight corsets has affected their eyesight. I don't quite understand how, but it doesn't signify. No one will believe it. If you really want to cure a woman of tight lacing, just persuade her to keep the measure of her abdomen on the same paper with her waist measure, and to watch her shoulder blades and the tip of her nose. Why, sometimes their shoulder blades get crowded up and stick out like rudimentary wings, and they aren't pretty with evening dresses.

